# Supplementary material for: Improved Mechanical, Thermal, and Hydrophobic Properties of PLA Modified with Alkoxysilanes by Reactive Extrusion Process
Source: Polymers (Basel). 2021 Jul 27;13(15):2475. doi: 10.3390/polym13152475 (PMC8347007; doi:10.3390/polym13152475)
Supplement: Supplementary file 1 [file polymers-13-02475-s001.zip › polymers-1298937 supplementary final c.pdf]

polymers

Supplementary Material: Improved Mechanical, Thermal and Hydrophobic Properties of PLA Modified with Alkoxysilanes by Reactive Extrusion Process

Elena Torres, Aide Gaona, Nadia García-Bosch, Miguel Muñoz, Vicent Fombuena, Rosana Moriana, Ana Vallés-Lluch

Improved mechanical and hydrophobic properties of PLA modified with alkoxysilanes by reactive extrusion process.

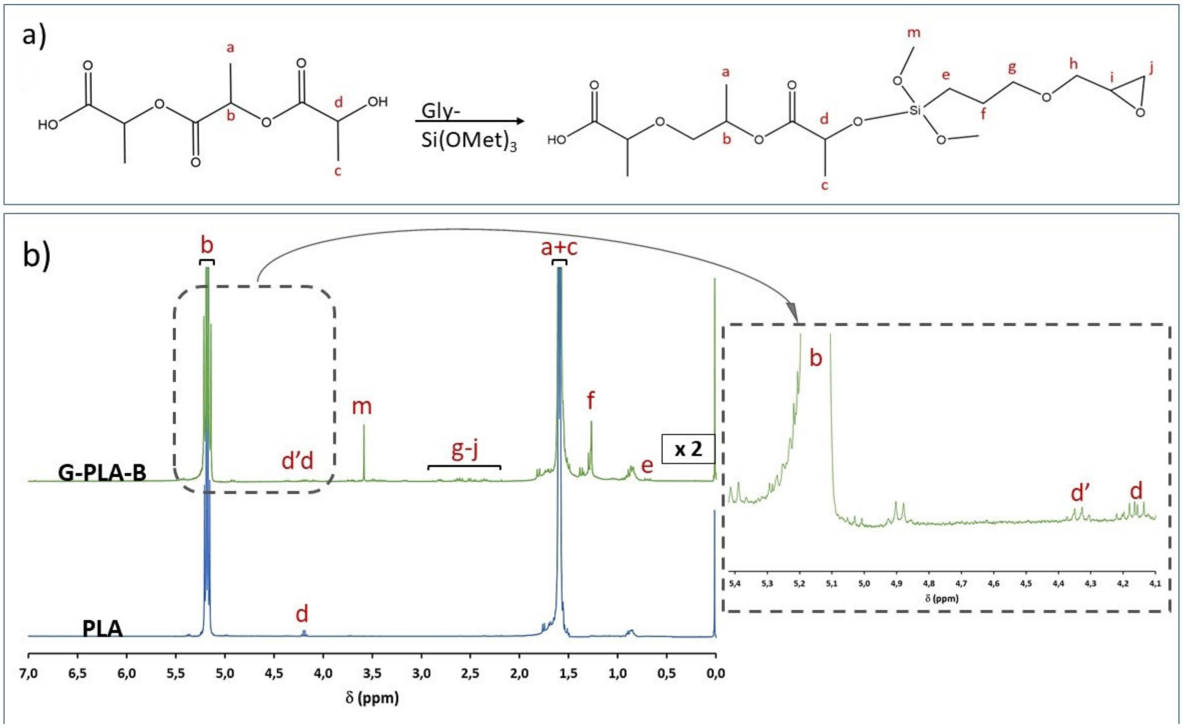

Figure S1. (**a**) Reaction scheme of the PLA modification with (3-glycidyloxypropyl)trimethoxysilane and (**b**)^{1}H NMR of raw and modified PLA different with concentration of (3-glycidyloxypropyl)trimethoxysilane: 1.3 and 2.7 wt%.

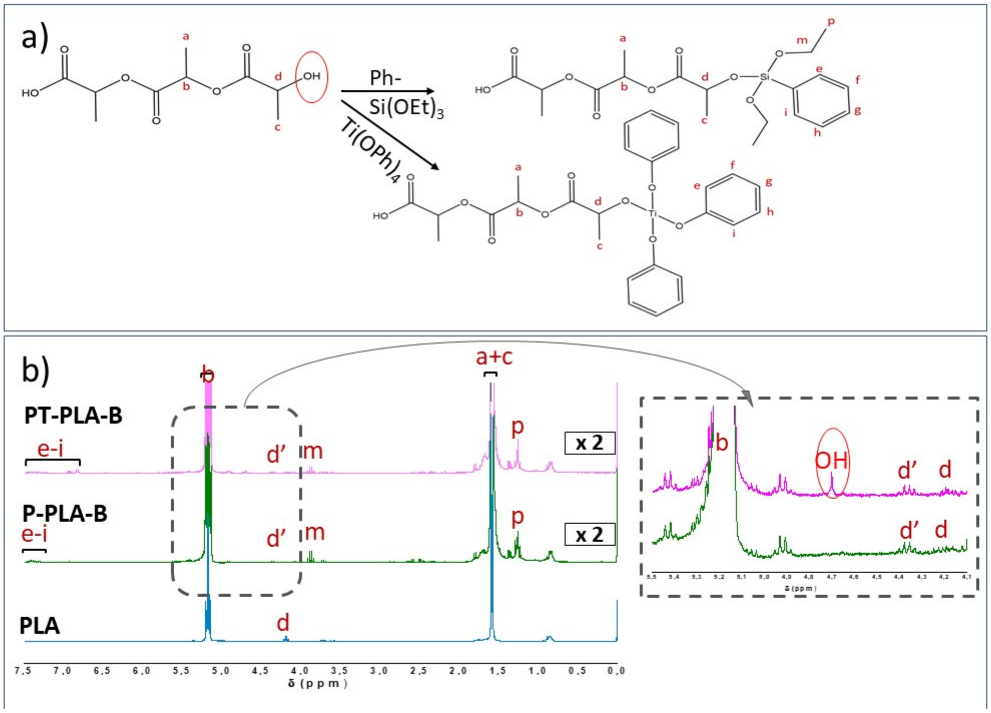

Figure S2. (**a**) Reaction scheme of the PLA modification with phenyltriethoxysilane and titanium phenoxide, (**b**)^{1}H NMR of raw and modified PLA with phenyltriethoxysilane and titanium phenoxide: P-PLA-B and PT-PLA-B.
